# Short-Term Serum-Free Culture Reveals that Inhibition of Gsk3β Induces the Tumor-Like Growth of Mouse Embryonic Stem Cells

**DOI:** 10.1371/journal.pone.0021355

**Published:** 2011-06-23

**Authors:** Yanzhen Li, Tamaki Yokohama-Tamaki, Tetsuya S. Tanaka

**Affiliations:** Department of Animal Sciences, Institute for Genomic Biology, University of Illinois at Urbana-Champaign, Urbana, Illinois, United States of America; University of Southern California, United States of America

## Abstract

Here, we present evidence that the tumor-like growth of mouse embryonic stem cells (mESCs) is suppressed by short-term serum-free culture, which is reversed by pharmacological inhibition of Gsk3β. Mouse ESCs maintained under standard conditions using fetal bovine serum (FBS) were cultured in a uniquely formulated chemically-defined serum-free (CDSF) medium, namely ESF7, for three passages before being subcutaneously transplanted into immunocompromised mice. Surprisingly, the mESCs failed to produce teratomas for up to six months, whereas mESCs maintained under standard conditions generated well-developed teratomas in five weeks. Mouse ESCs cultured under CDSF conditions maintained the expression of *Oct3/4*, Nanog, Sox2 and SSEA1, and differentiated into germ cells *in vivo*. In addition, when mESCs were cultured under CDSF conditions supplemented with FBS, or when the cells were cultured under CDSF conditions followed by standard culture conditions, they consistently developed into teratomas. Thus, these results validate that the pluripotency of mESCs was not compromised by CDSF conditions. Mouse ESCs cultured under CDSF conditions proliferated significantly more slowly than mESCs cultured under standard conditions, and were reminiscent of *Eras*-null mESCs. In fact, their slower proliferation was accompanied by the downregulation of *Eras* and *c-Myc*, which regulate the tumor-like growth of mESCs. Remarkably, when mESCs were cultured under CDSF conditions supplemented with a pharmacological inhibitor of Gsk3β, they efficiently proliferated and developed into teratomas without upregulation of *Eras* and *c-Myc*, whereas mESCs cultured under standard conditions expressed *Eras* and *c-Myc*. Although the role of Gsk3β in the self-renewal of ESCs has been established, it is suggested with these data that Gsk3β governs the tumor-like growth of mESCs by means of a mechanism different from the one to support the pluripotency of ESCs.

## Introduction

Embryonic stem cells (ESCs) [1], [2], [3] and induced pluripotent stem cells (iPSCs) [4], [5], [6], [7] are very promising tools for use in drug screening and customized tissue replacement [8] because they are capable of self-renewal that sustains pluripotency. The self-renewal and pluripotency of mouse stem cells (ESCs and iPSCs) are maintained by extrinsic factors, such as supplementing basal culture medium with leukemia inhibitory factor (LIF) [9], [10], [11], [12], [13] and fetal bovine serum (FBS). FBS further facilitates their self-renewal by offering other factors, such as bone morphogenetic protein 4 (Bmp4) [14], retinoids [15], [16], [17], threonine [18] and glutathione [19]. However, FBS also provides cultures with many other uncharacterized components that may affect the capability of ESCs and iPSCs to self-renew and differentiate. Undefined culture conditions using animal sera may have contributed to results finding contradictory roles of the Wnt signaling pathway in ESCs [20], [21], [22], [23]. However, it is now firmly established that pharmacological inhibition of glycogen synthase kinase 3β (Gsk3β) promotes the self-renewal of both mouse [22], [24] and human ESCs [23], and derivation of mouse ESCs [25].

To eliminate the effects of unknown components in animal sera as well as the contamination of animal products, chemically-defined serum-free culture methods have been established [14], [26], [27], [28]. Typically, defined culture media are composed of critical growth factors (e.g., LIF and/or Bmp4) and other factors present in animal sera, such as hormones (e.g., insulin and transferrin), vitamins, fatty acids and minerals. Commercially-made serum replacements that may contain these components in animal sera [29] are often used to maintain ESC culture (e.g., [30]), although the exact components cannot be disclosed by their patents [28]. The maintenance of the undifferentiated state of mouse ESCs (mESCs) using defined culture media has been well documented [31], [32]. Furthermore, the pluripotency of these mESCs has been validated by their differentiation *in vitro* [26], [32] or by the development of chimeric mice [14].

Another way to validate the pluripotency of ESCs and iPSCs is to examine the ability of these cells to develop into tumors called teratomas after their transplantation into immunocompromised mice [33], [34], [35]. Such teratoma formation assays have validated the pluripotency of mESCs maintained in the presence of a Gsk3β inhibitor [23], [25]. This method, which requires no special technique or equipment and reduces the use of experimental animals, is particularly useful and widely accepted for the validation of pluripotency in human ESCs and iPSCs [13], [36]. However, their tumor-like growth hampers the therapeutic application of human iPSCs [37]. Little is known about the inherent tumorigenic property of ESCs, except that the oncogene *Eras* regulates the tumor-like growth of mESCs via activation of Akt1 [38], which may result in inactivation of Gsk3β [24], [39], [40]. However, human ESCs do not express human *ERAS* [41], [42], but grow into teratomas [3]. Therefore, the underlying mechanism involved in the tumor-like growth of ESCs remains unknown.

On the other hand, mESCs contribute to the development of normal chimeras, instead of forming teratomas, when mixed with mouse preimplantation embryos. This finding indicates that mESCs require proper extrinsic signals or niches [43] to differentiate normally and to contribute to the development of chimeras. In contrast, mESCs exhibit cell death when they are cultured without LIF [26], [44]. This result raises the question of whether mESCs inherently possess the tumor-like property or are provided with extrinsic signals that promote their tumorigenesis. In this report, we present experimental evidence that short-term serum-free culture reduces the tumorigenicity of mESCs, which is reversed by pharmacological inhibition of Gsk3β. It is suggested with these data that the activity of Gsk3β orchestrates the tumor-like growth of ESCs, which may support a novel mechanism independent from the one regulating the pluripotency of ESCs.

## Results

### Mouse embryonic stem cells reduced their tumorigenicity but maintained their pluripotency under chemically-defined serum-free culture

**Figure 1:**
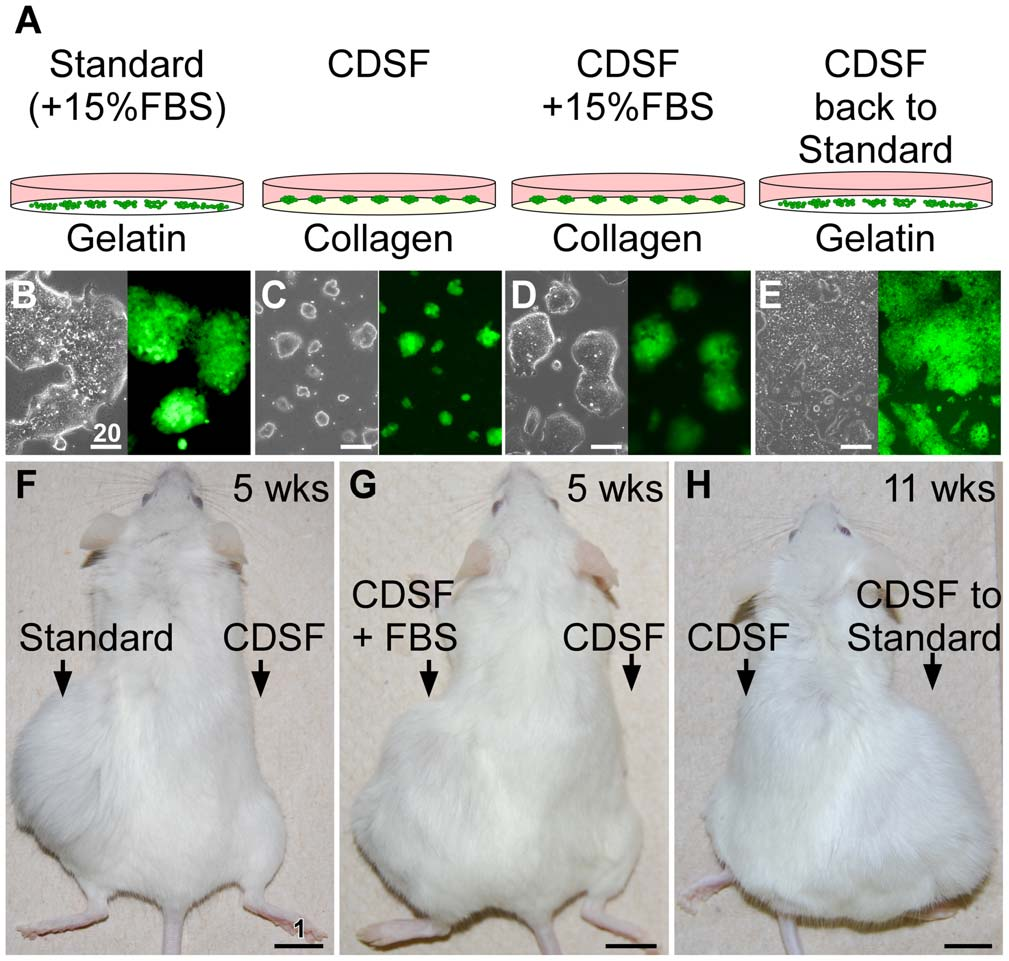
The tumorigenicity of mouse embryonic stem cells can be reduced by short-term serum-free culture. (A): A mouse embryonic stem cell (mESC) line harboring an EGFP reporter driven by the *Oct3/4* promoter (*Oct3/4::EGFP*) was maintained in either standard or chemically-defined serum-free (CDSF) medium as indicated. (B–E): Phase contrast (left) and fluorescence (right) images of the ESC line under the conditions indicated above are shown. Bars, 20 µm. (F–H): After the mESCs were cultured as indicated, they were transplanted into NOD-SCID mice subcutaneously. Teratoma formation was observed by week 11. Bars, 1 cm.

To examine whether mESCs are determined to grow as teratomas, we cultured mESCs in chemically-defined serum-free medium with LIF (referred to as “CDSF”) [26] for three passages (Fig. 1A, 1C) and subcutaneously transplanted them into non-obese diabetic mice with severe combined immunodeficiency disease (NOD-SCID mice) [45]. We used a uniquely formulated serum-free medium, ESF7 (see Materials and Methods), because components in this medium is fully disclosed [26]. This medium has been used to maintain mESCs by other studies (e.g., [46], [47]). However, the pluripotency of mESCs cultured under this medium has not been tested by teratoma formation assays. Surprisingly, these mESCs failed to grow as teratomas (Fig. 1F-1H), whereas mESCs maintained under standard conditions (Fig. 1A, 1B) grew into a well-developed teratoma in 5 weeks (Fig. 1F, 2A–2E, and supporting information Fig. S1A). When mESCs were cultured in CDSF supplemented with 15% FBS (referred to as “CDSF+FBS”; Fig. 1A, 1D), they formed a well-developed teratoma in 5 weeks (Fig. 1G, 2F–2J, and supporting information Fig. S1B). Thus, tumorigenesis in mESCs is not simply inhibited by CDSF conditions. When the injections were properly performed, we did not observe blood coming out of the injection sites. Only properly performed injections were counted in the present study.

**Figure 2:**
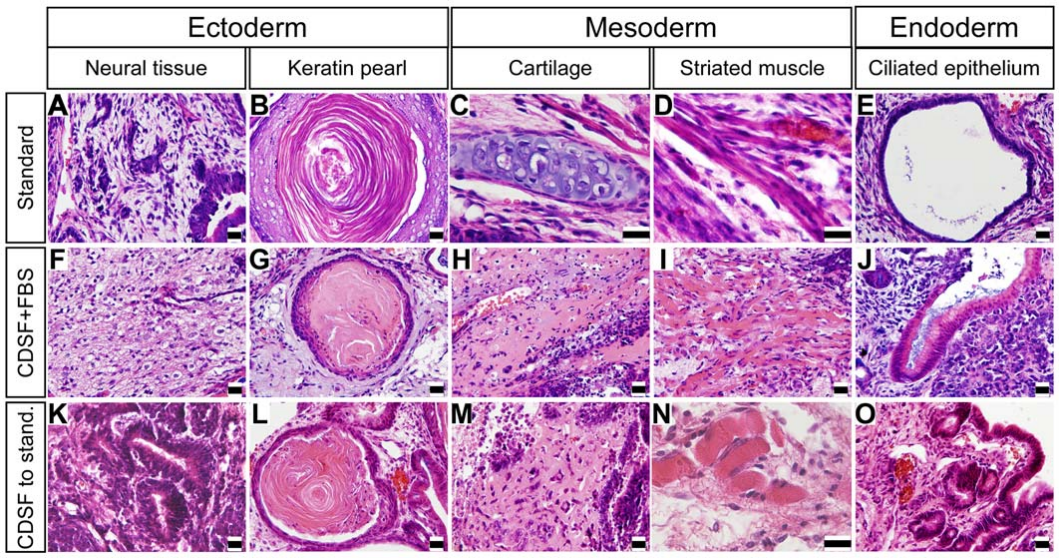
Identification of three germ layers in teratomas. (A–E): Teratomas developed from mESCs cultured under standard conditions. (F–J): Teratomas developed from mESCs cultured in CDSF supplemented with 15% FBS (CDSF+FBS). (K–O): Teratomas developed from mESCs cultured in CDSF followed by transfer to standard conditions (CDSF to Stand). Ectoderm is represented by neural tissue and keratin pearl, mesoderm is represented by cartilage and striated muscle, and endoderm is represented by ciliated epithelium. Bars, 20 µm.

**Figure 3:**
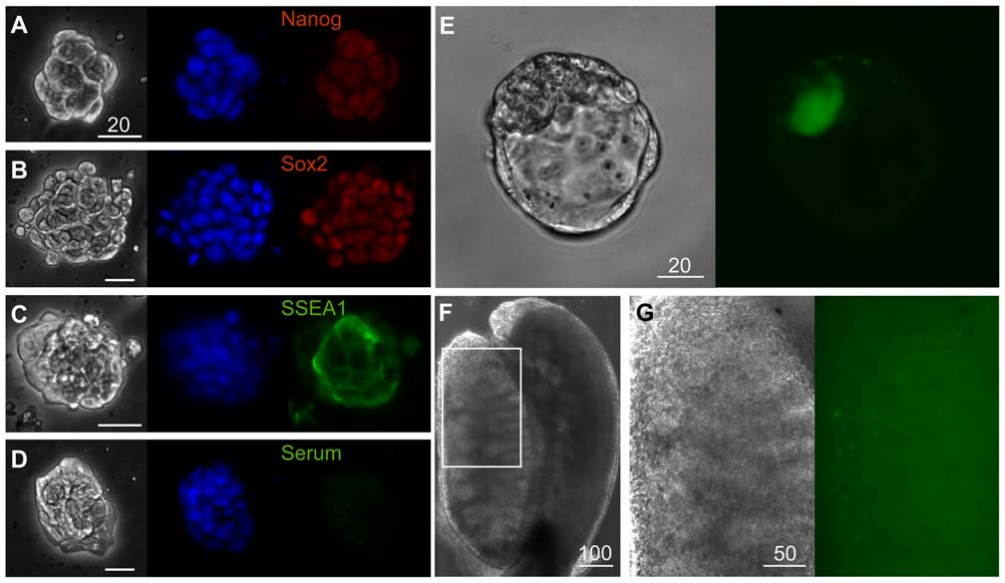
Mouse embryonic stem cells cultured under CDSF conditions are pluripotent. (A–D): Mouse ESCs cultured under CDSF conditions were immunostained (right) with either specific antibodies against Nanog (A), Sox2 (B) and SSEA1 (C), or normal mouse serum (D). Also, phase contrast (left), and DAPI-stained (middle) images of these mESCs are shown. Bars, 20 µm. (E): Phase contrast (left) and fluorescence (right) images of a chimeric blastocyst aggregated with the *Oct3/4::EGFP* mESC line. Bar, 20 µm. (F): A phase contrast image of a male gonad dissected from a chimeric E13.5 embryo. The rectangle indicates the area shown in G. Bar, 100 µm. (G): Enlarged phase contrast (left) and fluorescence (right) images of the male gonad shown in F. Bar, 50 µm.

We conclude that CDSF did not compromise the pluripotency of mESCs *per se* for the following reasons. First, throughout the culture period (three passages, 9–12 days), the transcriptional activity of the master regulator of pluripotency, *Oct3/4* (*Pou5f1*) [48], [49], [50], was validated using a mESC line that expresses the enhanced green fluorescent protein (EGFP) under the *Oct3/4* promoter [51] (Fig. 1B, 1C). Unless otherwise noted, this mESC line was used throughout the present study. Next, immunofluorescence microscopy confirmed that the mESC line cultured under CDSF conditions maintained the expression of Nanog, Sox2, and SSEA1 (Fig. 3A–3C). In addition, when the mESC line cultured under CDSF conditions was aggregated with wild-type morulae, resulting chimeric blastocysts exhibited green fluorescence in the inner cell mass (Fig. 3E). Eleven days after these blastocysts were transferred to pseudopregnant females, fluorescent cells were detected in a nascent male gonad of an embryo at embryonic day 13.5 (Fig. 3F, 3G).

Furthermore, when the mESC line was maintained in either CDSF+FBS (n = 4) or CDSF for three passages and brought back to the standard medium for one or two passages (referred to as “CDSF-Standard”, n = 4; Fig. 1A), EGFP expression was maintained (Fig. 1D, 1E) and the cells became teratomas (Fig. 1G, 1H, and 2F–2O; *p*<0.00016, see supporting information Table S1 and Fig. S1B, S1C). Similar results were obtained using four different lots of FBS. These data demonstrate that tumorigenesis in mESCs can be suppressed by short-term culture in the serum-free medium. The plasticity of tumorigenicity in mESCs appears to be unique; embryonic carcinomas (F9) [52], which are also pluripotent stem cells, formed teratomas when cultured in CDSF and transplanted (supporting information Fig. S1D). Likewise, germline-incompetent mESCs, D3, grew into teratomas (data not shown).

### Mouse embryonic stem cells cultured under serum-free conditions exhibited longer doubling time while maintained expression of genes associated with cellular pluripotency

**Figure 4:**
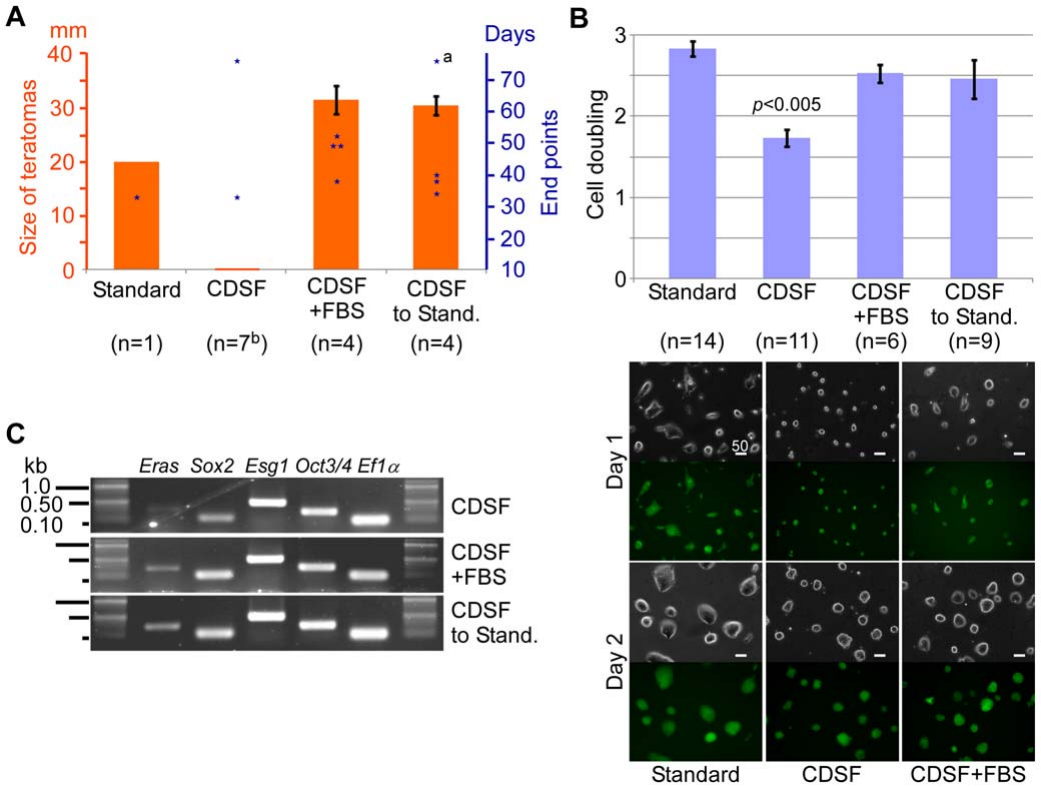
Phenotypic changes observed in mouse embryonic stem cells cultured under CDSF conditions. (A): The sizes of the teratomas formed (orange bar, left axis) and the number of days required for the experimental NOD-SCID mice to reach their end points (blue stars, right axis) were compared among mESCs cultured under the conditions indicated (see Fig. 1A). Parentheses indicate the number of biological replicates (i.e., mESCs prepared at different passages) per culture condition. Standard errors of the means are indicated by bars. ^a^: Only one passage in standard conditions followed CDSF culture. ^b^: Two out of seven transplantations showed no sign of teratoma formation when paired with the standard and CDSF-Standard conditions, whereas five out of seven transplantations showed no sign of teratoma formation for 6 months. (B, top): Cell doublings were measured every 48 hours after plating 1×10^6^ cells per well onto 6-well plates (see Materials and Methods for the formula). Only CDSF conditions produced statistically-significant differences compared to standard conditions. Parentheses indicate the number of biological replicates per condition. Standard errors of the means are indicated by bars. (B, bottom): Phase contrast (top) and fluorescence (bottom) images are shown for the *Oct3/4::EGFP* mESC line (Fig. 1A) grown under the conditions indicated below 1 and 2 days after plating 0.1×10^6^ cells per well in 6-well plates. Bars, 50 µm. (C): Abundance of each transcript indicated above was examined in mESCs cultured under each condition on the right by 25 cycles of PCR. *Ef1α* was used as a reference.

When NOD-SCID mice transplanted with mESCs reached their end points, they were sacrificed and examined for teratomas. This procedure usually yielded teratomas of about 30 mm in diameter (bars in Fig. 4A). However, the number of days needed for the experimental animals to reach their end points varied (stars in Fig. 4A). Interestingly, it took 47±3.1 days for mESCs cultured in CDSF+FBS to grow into 30 mm teratomas, whereas it took 37±1.8 days for mESCs cultured in CDSF followed by standard conditions for two passages to reach 30 mm in diameter. However, when mESCs cultured in CDSF were passaged only once into standard conditions, it took 76 days for these mESCs to grow into a 30 mm teratoma. Although this sample size is too small to be statistically significant, it is suggested with these data that CDSF may even suppress the growth of teratomas.

Among the experimental NOD-SCID mice examined in this study, two out of the seven mice had mESCs cultured in CDSF injected into one side of the animal and mESCs cultured in media containing FBS injected into the other side. Therefore, the formation of teratomas from mESCs cultured in CDSF could not be examined beyond the end point of the animals (Fig. 4A). However, in the other five animals, we were able to determine that transplanted mESCs under CDSF did not generate teratomas for up to 6 months.

At one injection site, we were able to identify a tiny mass of mESCs that had been cultured under CDSF-Standard conditions one week after transplantation (supporting information Fig. S1E). The mESCs in this mass had the appearance of cells undergoing initial differentiation (supporting information Fig. S2). However, we did not observe any cellular mass at the injection sites that were derived from mESCs cultured in CDSF at either one week or six months after transplantation.

Mouse ESCs cultured in CDSF exhibited a significantly longer doubling time (∼28 hrs) than ones cultured under standard conditions (∼17 hrs, *p*<0.005) during the first two passages, whereas ones cultured in CDSF+FBS or under CDSF-Standard conditions took ∼19 and ∼20 hrs to divide, respectively (Fig. 4B, top). Similar results were obtained with another mESC line, W4 [53], [54] (data not shown). The differences in doubling times of mESCs were evident as soon as two days after transfer to CDSF conditions (1.91 doublings ± S.E.M. of 0.0967 vs. 2.72 doublings ± S.E.M. of 0.0923 for the standard condition in 48 hrs, *p*<0.005). Despite the longer doubling time, mESCs cultured in CDSF did proliferate steadily (Fig. 4B, bottom). Additionally, transcripts associated with cellular pluripotency, *Sox2*, *Esg1*/*Dppa5* and *Oct3/4*, were expressed in mESCs cultured in CDSF (Fig. 4C). Interestingly, *Eras*, which regulates the tumorigenic growth of mESCs [38], was downregulated in these mESCs (Fig. 4C), whereas it became upregulated when mESCs were maintained in CDSF+FBS or under CDSF-Standard conditions (Fig. 4C, see also supporting information Fig. S5). Collectively, the loss of tumor-like potential in mESCs cultured under CDSF is associated with a slower growth rate and the reduced expression of *Eras*. It is interesting to note that *Eras*-null mESCs can contribute to the germline in chimeric animals but show significantly reduced growth rate [38].

### Pharmacological inhibition of Gsk3β reversed the effect of serum-free culture on the tumor-like growth of mouse embryonic stem cells

To identify a potential serum factor responsible for inducing the tumor-like growth in mESCs, initially we focused on molecules known to sustain the self-renewing growth of mESCs, such as LIF [9], [10], Bmp4 [14], vitamin A derivatives (all-trans retinoic acid, RA [15], and retinol [16], [17]), and simultaneous inhibition of Erk and Gsk3β [31]. Pharmacological inhibition of Gsk3β alone promotes self-renewal of both mouse and human ESCs [23]. We excluded LIF from screening because CDSF contains LIF [26]. In the absence of LIF, mESCs undergo differentiation or cell death [26], [44]. We also ruled out an inhibitor of Erk [31], which acts downstream of FGF receptors, because inhibition of Erk cannot promote the growth of mESCs [31]. Therefore, we focused on testing other molecules such as Bmp4, RA, retinol with or without retinol binding protein (RBP) [55], and an inhibitor of Gsk3β (CIHR99021) [31].

**Figure 5:**
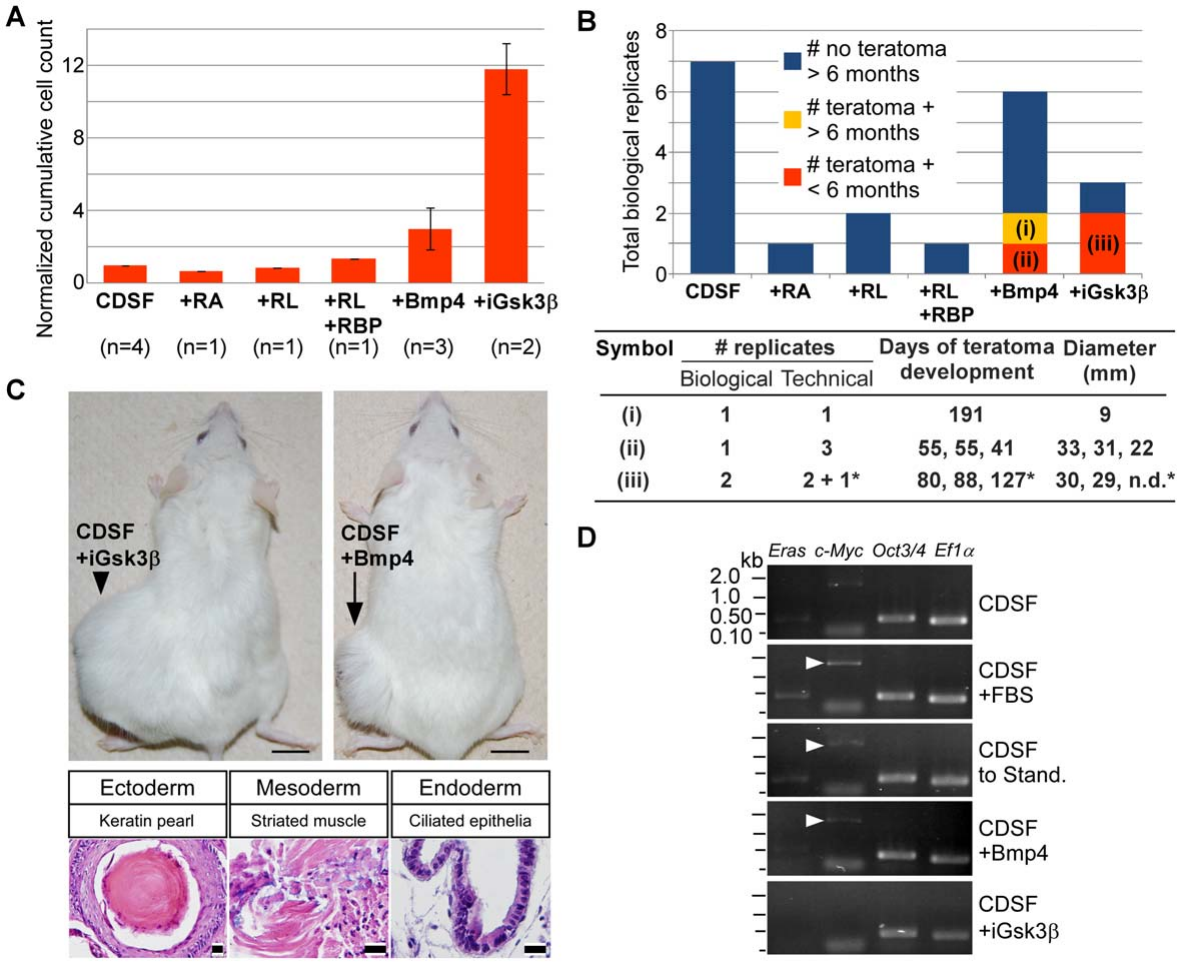
Screening of factors responsible for the tumor-like growth of mouse embryonic stem cells. (A): Cumulative numbers of mESCs were compared among ESCs cultured under each condition indicated for 3 passages. Cell counts were normalized to CDSF conditions. A value for CDSF conditions is normalized to 1. Parentheses indicate the number of biological replicates per condition. Standard errors of the means are indicated by bars. +RA, CDSF with retinoic acid; +RL, CDSF with retinol; +RL+RBP, CDSF with retinol and retinol binding protein; +Bmp4, CDSF with Bmp4; +iGsk3β, CDSF with the Gsk3β inhibitor. (B): Total numbers of biological replicates that resulted in formation of teratomas were compared among mESCs cultured under each condition indicated. Orange and yellow boxes indicate the number of biological replicates that developed into teratomas within 6 months and in more than 6 months, respectively. Blue bars indicate the number of biological replicates that failed to form teratomas for more than 6 months. Data for the Gsk3β inhibitor include results obtained with R1 and W4 mESCs. For those biological replicates indicated as (i), (ii) and (iii) in the bar chart, the number of technical replicates, the number of days needed for the experimental animals to reach their end points, and the sizes of the longest axis of the teratomas are shown per technical replicate. Asterisks (*) indicate that W4 mESCs were transplanted. n.d., not determined. (C, top): NOD-SCID mice that received transplantation of mESCs cultured in CDSF supplemented with Bmp4 (right) or the Gsk3β inhibitor (left) are shown. Bars, 1 cm. (C, bottom): Representative histological images are shown. The presence of three germ layers (see the legend for Fig. 2) is evident in teratomas developed from mESCs cultured in CDSF supplemented with the Gsk3β inhibitor. Bars, 20 µm. (D): Abundance of each transcript indicated above was examined in mESCs cultured under each condition on the right by 24 cycles of PCR. *Ef1α* was used as a reference. White arrowheads indicate the PCR product of *c-Myc*.

Addition of RA in CDSF induced differentiation of mESCs as evidenced by the reduced expression level of EGFP (supporting information Fig. S3A). Addition of retinol with or without RBP in CDSF did not induce differentiation of mESCs (supporting information Fig. S3B, S3C), but failed to accelerate their growth and to induce teratoma formation (Fig. 5A, 5B). In contrast, mESCs cultured in CDSF with Bmp4 or the Gsk3β inhibitor maintained *Oct3/4* expression (supporting information Fig. S3D, S3E), increased the number of cell doublings (Fig. 5A) and formed teratomas in 17% or 67% of transplantations by 7 months, respectively (Fig. 5B, 5C and supporting information Fig. S1F, S1G). When cultured in other established CDSF media supplemented with N2 [56], B27 [57], and either Bmp4 and LIF [14], or pharmacological inhibitors of Erk and Gsk3β [22], mESCs grew into teratomas more efficiently (see “N2B27-BL” and “N2B27-2i” in supporting information Table S1 and Fig. S4). Also, W4 mESCs exhibited similar phenotypic changes when maintained in CDSF with the Gsk3β inhibitor (Fig. 5B). Gsk3β is known to regulate the activity of the c-Myc protein in mESCs [24], [58]. However, RT-PCR analysis showed that inhibition of Gsk3β did not result in upregulation of *Eras* and *c-Myc* in mESCs cultured in CDSF, whereas Bmp4 induced upregulation of *c-Myc* (Fig. 5D and supporting information Fig. S5).

## Discussion

In this report, we present experimental evidence to suggest that short-term CDSF culture reduces the tumor-like growth of mESCs, which is reversed by pharmacological inhibition of Gsk3β. Our present study indicates that downstream of Gsk3β is primarily responsible for tumorigenesis in mESCs, which may involve uncharacterized gene products. Although the exact mechanism currently remains unknown, our present study provides a basis for further study to establish the signaling pathway responsible for the tumor-like property of ESCs.

In general, serum provides hormones, growth factors, and steroids to cultured cells. It also contains remnants of plasma components used for the activation and processing of blood clots and substances that do not normally pass through the endothelial barrier [59], [60], [61]. Therefore, serum is similar but not identical to the interstitial fluid (i.e., lymph) that surrounds cells *in vivo* [62]. We were unable to observe any cellular mass at the injection sites that were derived from mESCs cultured in CDSF at either one week or six months after transplantation. Therefore, it is suggested with our data that interstitial fluid will not support the tumor-like growth of mESCs.

Perhaps mESCs cultured under short-term CDSF conditions became more susceptible to LIF and exhibited cell death after transplantation due to the absence of a continuous supply of LIF [26]. However, because each mESC line shows a different degree of LIF dependency [54], other mouse pluripotent stem cells cultured in the serum-free medium may exhibit a capability to continuously grow after transplantation, as we have seen in embryonic carcinomas (F9; supporting information Fig. S1D) and germline-incompetent mESCs, D3 (data not shown). In addition, we found that the formation of teratomas became sporadic when we transplanted 0.5×10^6^ mESCs cultured under standard conditions for a period of 6 months. Collectively, our study demonstrated that one or more extrinsic factors or niche [43] plays an important role in the formation of a teratoma. This idea is further supported by the fact that two mESCs were sufficiently able to grow into a teratoma only when mixed with 2×10^6^ non-tumorigenic fibroblasts (MRC-5) prior to transplantation into immunocompromised mice [63]. Interestingly, the slow growth observed in *Eras*-null mESCs became more evident when they were cultured without feeder cells [38].

Our present data indicate that animal sera contain one or more factors that inhibit the activity of the Gsk3β protein (Fig. 1, 4 and 5). Gsk3β is involved in the canonical Wnt signaling pathway [64], [65], [66] and interacts with other biologically important signaling pathways such as phosphoinositide 3-kinase (PI3K)-Akt1 [40], [67], [68], Bmp4 [69] and hedgehog [70], [71], [72] signaling pathways. Although the secreted protein Wnt eventually inhibits the activity of Gsk3β, the role of Wnt in the maintenance of self-renewal and pluripotency of ESCs remains elusive [20], [21], [22], [23]. On the other hand, pharmacological inhibition of Gsk3β supports the self-renewal and pluripotency of ESCs [22], [23], [24], [25]. Now, the question is what Gsk3β upstream and downstream genes are in self-renewing ESCs.

Both LIF-Stat3 [73], [74] and insulin pathways activate the PI3K-Akt1 signaling pathway [24], [68], which mediates the inactivation of Gsk3β [24], [40], [64]. However, CDSF includes LIF and insulin [26], and failed to support the tumor-like growth of mESCs (Fig. 1 and 4). Thus, LIF and insulin are not the upstream of Gsk3β. Because Eras activates Akt1 [38], the downregulation of *Eras* in mESCs cultured in CDSF (Fig. 4C and 5D) may have resulted in the activation of Gsk3β. However, human ESCs do not express human *ERAS* but they do grow into teratomas [41], [42]. Because this signaling cascade is not evolutionarily conserved, Eras is not the upstream of Gsk3β.

Based on our results, Bmp4 poorly promoted proliferation and teratoma formation of mESCs cultured under CDSF conditions (Fig. 5A, 5B). It is well known that the Bmp4 and Wnt signals interact with each other in many morphogenetic events, which could result in either synergistic or antagonistic effects depending on cell types [69]. Because of the following two observations, we consider the effect of Bmp4 on Gsk3β or the tumor-like growth of mESCs antagonistic or indirect. First, our RT-PCR results revealed that mESCs cultured in CDSF supplemented with Bmp4 upregulated *c-Myc*, which was not the case in mESCs treated with the Gsk3β inhibitor (Fig. 5D and supporting information Fig. S5). Second, in one set of our experiments, Bmp4 efficiently induced formation of teratomas when ESCs at the earlier passage number (passage 7) were used for the culture (see (ii) in Fig. 5B). In contrast, when mESCs at passage 9 or later were cultured in CDSF with Bmp4 and transplanted, they sporadically developed into teratomas. Further investigation is required to determine the upstream of Gsk3β in ESCs.

We showed that a CDSF medium supplemented with Bmp4 and LIF supported the proliferation of mESCs that maintained transcriptional activity of *Oct3/4* (supporting information Fig. S3D). In contrast, Hayashi *et al*. demonstrated that a CDSF medium supplemented with Bmp4 supported the differentiation of trophoblasts from mESCs [47]. However, Hayashi *et al*. used a basal CDSF medium that lacked oleic acid and LIF, and plated mESCs on laminin-coated dishes, but not on collagen IA-coated dishes. In addition, the presence of LIF under these CDSF conditions inhibited the differentiation of trophoblasts [47]. Therefore, it is likely that the different chemical composition of a basal medium contributed to the differentiation of trophoblasts when Bmp4 was supplemented in the culture.

It is interesting to note that in concert with LIF, Bmp4 suppresses differentiation of the neural lineage in mESCs maintained under serum-free conditions [14] supplemented with N2 and B27, which were originally developed to culture a neuroblastoma cell line [56] and hippocampal neurons [57], respectively. Mouse ESCs maintained under serum-free conditions supplemented with LIF, Bmp4, N2 and B27 are pluripotent and can contribute to the germline in chimeric animals [14], and grew into teratomas (supporting information Table S1 and Fig. S4). On the other hand, we showed that both the CDSF medium used in the present study, which contains LIF, and the CDSF medium supplemented with Bmp4 poorly sustained the tumor-like growth of mESCs. Taken together, it is suggested with these data that the cellular pluripotency and the tumor-like growth of ESCs may be regulated by different mechanisms, and that extrinsic factors play significant roles in cell fate decisions of mESCs. It will be interesting to investigate whether ESCs need unique substrate stiffness to grow into teratomas, because our previous study showed that stiffer substrates promoted differentiation of mESCs [75].

Gsk3β inhibits the activity of its target c-Myc [24], [76], which is involved in the self-renewal of mESCs [58] and responsible for an age-associated incidence of tumorigenesis in chimeric mice generated with mouse iPSCs [77], [78]. In contrast, our RT-PCR results showed that mESCs cultured under CDSF conditions with the Gsk3β inhibitor failed to significantly upregulate transcription of *Eras* and *c-Myc* (Fig. 5D) but efficiently developed into teratomas (Fig. 5B, 5C). Thus, we propose that Gsk3β downstream genes other than *c-Myc* may regulate the tumor-like growth of ESCs, which play independent roles from maintaining the self-renewal of ESCs. In addition, candidate Gsk3β downstream genes responsible for tumorigenesis in mESCs may act independently from ERAS in human ESCs [41], [42]. Currently, our investigation is ongoing. Further study to identify the candidate genes and to test their roles in human ESCs and iPSCs will lead us to establishing a strategy to significantly improve the safety of human iPSCs.

## Materials and Methods

### Ethics statement

Use of animals was approved by the Illinois Institutional Animal Care and Use Committee (protocol # 10093, approved on 6/18/10).

### Cell culture

A mouse embryonic stem cell (mESC) line of R1 [79], which expresses EGFP under the *Oct3/4* promoter [80], was kindly provided by Dr. William L. Stanford [51]. This *Oct3/4::EGFP* mESC line was thawed on feeders and maintained under standard [81] or chemically-defined serum-free (CDSF) [26] conditions at 37°C, 5% CO_2_. The exact number of passages that this mESC line has gone through is unknown. However, when these ESCs were brought to our laboratory, they were passaged on feeders two more times and frozen as a stock. When these ESCs were thawed on feeders, more frozen stocks were made at passage 5–7. These stocks were used for the current study. Under standard conditions, mESCs were maintained on 0.1% gelatin (Sigma-Aldrich, St. Louis, MO, http://www.sigmaaldrich.com)-coated tissue culture dishes in high glucose-Dulbecco's modified Eagle's medium (DMEM; Invitrogen, Carlsbad, CA, http://www.invitrogen.com) supplemented with 15% fetal bovine serum (FBS; Invitrogen), 0.1 mM non-essential amino acids (Invitrogen), 2 mM GlutaMax I (Invitrogen), 1 mM sodium pyruvate (Invitrogen), 100 U/ml penicillin and 0.1 mg/ml streptomycin (Sigma-Aldrich), 0.1 mM 2-mercaptoethanol (Sigma-Aldrich), and 1,000 U/ml LIF (Millipore, Billerica, MA, http://www.millipore.com). When the mESCs reached 80% to 100% confluence, they were routinely passaged at a ratio of 1∶6 every two days using TrypLETM Express (Invitrogen). The cells were discarded after being passaged 10 times onto gelatin-coated dishes. Approximately 1×10^5^/cm^2^ mESCs maintained under standard conditions were plated onto tissue culture dishes coated with 0.15 mg/ml type IA collagen (Nitta Gelatin Co., Osaka, Japan, http://www.nitta-gelatin.co.jp), which contain the ESF7 medium (Cell Science & Technology Institute Inc. Miyagi, Japan, http://www.cstimedia.com) [26] supplemented with 1,000 U/ml LIF. This was counted as passage 1 under CDSF conditions. Mouse ESCs at passage 7–12 were used to start CDSF culture. Mouse ESCs grown under CDSF conditions were split every 3 or 4 days with 0.02% EDTA (Sigma-Aldrich). Similarly, mESCs of W4 (129S6, purchased at passage 9; Taconic, Hudson, NY, http://www.taconic.com) and D3 (129S2/SvPas, CRL-1934, ATCC, Manassas, VA) were used to test CDSF conditions. When CDSF culture was supplemented with FBS, 0.02% EDTA was used for passaging cells. Following serum lots were used to supplement CDSF culture: Lot. 1359246 and 726570, Invitrogen; Lot. L0228, Atlanta Biologicals (Lawrenceville, GA, http://www.atlantabio.com); Lot. A74B00Z, Gemini Bio-Products (West Sacramento, CA, http://www.gembio.com). Images of cell morphology and fluorescence were taken under the same conditions using an inverted microscope equipped with an epi-fluorescence lamp (DMI4000B, Leica Microsystems, Wetzlar, Germany, http://www.leica-microsystems.com) [82], [83]. To measure the frequency of cell doubling, the mESCs were plated at 1×10^6^ per one well of 6-well plates. Two days after plating, the number of cells was counted for the second and third passages. Cell doubling was calculated based on the following formula: Cell doubling  =  log_2_[(the number of cells 2 days after plating)/(1×10^6^)]. Statistical tests were performed using the Mann-Whitney's U-test.

To compare the incidence of teratoma formation, another basal culture medium was prepared by mixing the NeurobasalTM medium supplemented with B27 (Invitrogen) 1:1 with DMEM/F12 (Invitrogen) supplemented with N2 (Invitrogen) and 50 µg/ml bovine serum albumin as described already [84]. The resulting basal medium was supplemented with either 10 ng/ml LIF and 10 ng/ml Bmp4 (R & D systems) [14], or 1 µM Stemolecule TM PD0325901 and 3 µM Stemolecule TM CHIR99021 (2i; Stemgent, Cambridge, MA, https://www.stemgent.com/) [22], and used to culture mESCs for three passages, which needed seven days before subcutaneous injection into NOD-SCID mice.

### Cell transplantation

At the fourth passage under standard or CDSF conditions, the mESCs were trypsinized and counted. TrypLETM Express was inactivated with an equal volume of 1 mg/ml soybean trypsin inhibitor (Sigma-Aldrich). For this purpose, no culture medium with animal serum was used. One to two million cells were centrifuged at 1,000 g for 5 min and resuspended into 25 µl PBS, which was mixed with 25 µl of 0.3 mg/ml type IA collagen. Mouse ESCs were kept on ice before being injected into NOD-SCID mice (the Jackson Laboratory, Bar Harbor, ME, http://www.jax.org) subcutaneously. Animal health was monitored routinely until the diameter of the tumors reached several few centimeters at which time the animals reached their end points and were euthanized. This procedure was approved by the Illinois Institutional Animal Care and Use Committee. The incidence of teratoma formation was statistically validated using the Fisher's exact probability test.

### Histology

Before dissecting the teratomas, pictures of the experimental animals were taken. The sizes of the longest axis of the teratomas were measured. Then, the teratomas were surgically dissected out, cut into smaller pieces, and fixed in 4% paraformaldehyde (Sigma-Aldrich) at 4°C overnight, followed by dehydration and embedding in Paraplast plus (Sigma-Aldrich). Sections of 8 µm thickness were cut and processed for standard hematoxylin and eosin staining.

### Immunofluorescence microscopy

The *Oct3/4::EGFP* ESC line was immunostained essentially as described previously [75], [82]. Goat anti-mouse Nanog (AF2729, R & D systems) and anti-human Sox2 (sc-17320, Santa Cruz Biotechnology, Santa Cruz, CA) polyclonal antibodies and a mouse anti-SSEA1 monoclonal antibody (Developmental Studies Hybridoma Bank, University of Iowa) were used as primary antibodies. Alexa Fluor 488 goat anti-mouse IgG (H+L) and Alexa Fluor 546 donkey anti-goat IgG (H+L) polyclonal antibodies (Invitrogen) were used as secondary antibodies. Fluorescence images were taken using the same exposure time (750 msec for 505 nm and 850 msec for 595 nm) and enhanced in the same way.

### Aggregation chimeras

Mouse ESCs cultured under CDSF conditions for 10–12 days were aggregated with zona-free morulae, which were obtained from superovulated C57BL/6 females (the Jackson Laboratory) mated with C57BL/6 males. Aggregated embryos were cultured in the KSOM medium with 1/2 amino acids (Millipore) for 14–15 hrs as described previously [79]. Chimeric blastocysts were transferred to pseudopregnant females at the transgenic mouse facility on campus.

### RT-PCR

A total of 1.6 µg of total RNA extracted from mESCs cultured under each condition was used to synthesize the first cDNA strand as previously described [83], [85]. PCR mixtures were prepared using Phusion DNA polymerase (New England Biolab, Ipswich, MA, http://www.neb.com) according to the manufacturer's instructions. The PCR conditions were as follows: initial denaturing at 98°C for 1 min followed by 19, 21, 23, 24 or 25 cycles of denaturing at 98°C for 10 sec, annealing at 65°C for 30 sec, extension at 72°C for 30 sec, and a final extension at 72°C for 7.5 min. The primer pairs have previously been described: *Eras* [38], *Oct4* [86], *Esg1* and *Ef1α* [87], *Sox2* [88], and *c-Myc* [89].

### Screening of factors

A day after mESCs were plated under CDSF conditions, these cultures were supplemented with 10 nM retinol (Sigma-Aldrich) in 100% EtOH with or without 1 nM retinol binding protein (RBP) from human urine (Sigma-Aldrich), 10 nM all-trans retinoic acid (Sigma-Aldrich), 10 ng/ml Bmp4 (R & D Systems, Minneapolis, MN, http://www.rndsystems.com), or 3 µM Stemolecule TM CHIR99021 in DMSO (Stemgent). Before applying RBP to culture, buffer exchange was carried out with Amicon Ultra (Millipore) to remove a preservative. Mouse ESCs grown under these conditions were split with 0.02% EDTA for 3 passages when they reached confluence. At the fourth passage, these mESCs were injected into NOD-SCID mice as described above.

## Supporting Information

Figure S1 **Anatomical images of NOD-SCID mice transplanted with mouse embryonic stem cells.** (A-D, F and G): Teratomas developed from mouse embryonic stem cells (mESCs) and embryonic carcinomas (F9 in D) cultured under the conditions indicated are shown. Also, the number of weeks (wks) needed for the experimental animals to reach their end points are shown. Bars, 1cm. CDSF+FBS, CDSF culture supplemented with fetal bovine serum; CDSF-Standard, CDSF conditions followed by standard conditions; CDSF (F9), embryonic carcinoma cells F9 maintained under CDSF conditions; CDSF+Bmp4, CDSF culture supplemented with Bmp4; CDSF+iGsk3β, CDSF culture supplemented with the Gsk3β inhibitor. (E): This animal was sacrificed one week after mESCs cultured under CDSF conditions followed by standard conditions were transplanted. The rectangle indicates the area shown in the inset. Bar, 1cm. The inset shows an enlarged image of a tiny mass of the mESCs depicted by dashed lines with a scale bar of 0.1 cm. See supporting information Fig. S2 for detail.
(TIF)

Figure S2 **Mouse embryonic stem cells exhibited initial differentiation as early as one week after transplantation when cultured under CDSF-Standard conditions.** Mouse ESCs were cultured in CDSF for three passages followed by transfer to standard conditions for two passages prior to transplantation. (A): An epithelialized cellular mass has aggregates formed among the collagen fibers (stained pale pink) used to transplant the mESCs. Rectangles indicate the areas shown in B, C and D. Bar, 500 µm. (B-D): Two types of cells are prominent, one of which is reminiscent of keratin pearls (B and D), and the other that resembles cartilage (C and D). Bars, 20 µm.
(TIF)

Figure S3 **Identification of factors that support the tumor-like growth of mouse embryonic stem cells maintained under CDSF conditions.** (A-E): Phase contrast (top) and fluorescence (bottom) images of mESCs under CDSF conditions supplemented with each factor indicated above are shown. Bars, 20 µm. CDSF+RA, CDSF with retinoic acid; CDSF+RL, CDSF with retinol; CDSF+RL+RBP, CDSF with retinol and retinol binding protein; CDSF+Bmp4, CDSF with Bmp4; CDSF+iGsk3β, CDSF with the Gsk3β inhibitor.
(TIF)

Figure S4 **The incidence of teratoma formation was compared among different CDSF conditions.** The sizes of the teratomas formed (orange bar, left axis) and the number of days required for the experimental NOD-SCID mice to reach their end points (blue stars, right axis) were compared among mESCs cultured under the conditions indicated. Parentheses indicate the number of biological replicates (i.e., mESCs prepared at different passages) per culture condition. Standard errors of the means are indicated by bars. Two transplantations for ESF7 showed no sign of teratoma formation when paired with the standard conditions. Standard, Standard conditions; CDSF, CDSF conditions; BL, other established CDSF conditions supplemented with N2, B27, Bmp4 and LIF; 2i, other established CDSF conditions supplemented with N2, B27, and pharmacological inhibitors of Erk and Gsk3β.
(TIF)

Figure S5 **Expression levels of markers were compared among different culture conditions.** Abundance of each transcript indicated above was examined in mESCs cultured under each condition on the right by 19, 21 and 23 cycles of PCR (indicated by triangles). *Ef1α* was used as a reference. White arrowheads indicate the PCR product of *c-Myc*.
(TIF)

Table S1 **Effects of serum on the tumorigenicity of mouse embryonic stem cells.**
(DOCX)
