# Supplementary figures and images for: Short-Term Serum-Free Culture Reveals that Inhibition of Gsk3β Induces the Tumor-Like Growth of Mouse Embryonic Stem Cells
Source: PLoS One. 2011 Jun 23;6(6):e21355. doi: 10.1371/journal.pone.0021355 (PMC3121758; doi:10.1371/journal.pone.0021355)

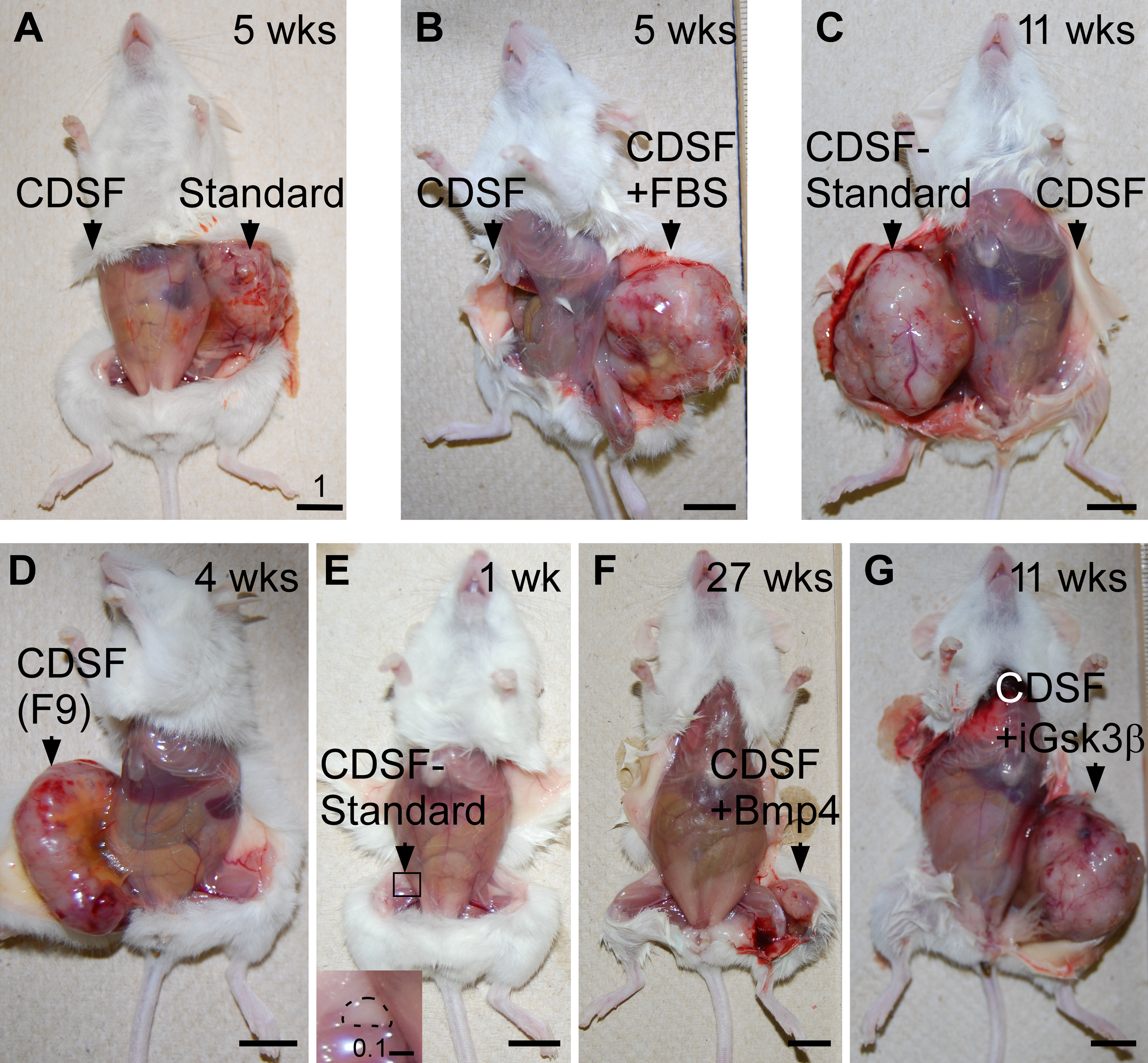

Supplement: Figure S1 — Anatomical images of NOD-SCID mice transplanted with mouse embryonic stem cells. (A-D, F and G): Teratomas developed from mouse embryonic stem cells (mESCs) and embryonic carcinomas (F9 in D) cultured under the conditions indicated are shown. Also, the number of weeks (wks) needed for the experimental animals to reach their end points are shown. Bars, 1cm. CDSF+FBS, CDSF culture supplemented with fetal bovine serum; CDSF-Standard, CDSF conditions followed by standard conditions; CDSF (F9), embryonic carcinoma cells F9 maintained under CDSF conditions; CDSF+Bmp4, CDSF culture supplemented with Bmp4; CDSF+iGsk3β, CDSF culture supplemented with the Gsk3β inhibitor. (E): This animal was sacrificed one week after mESCs cultured under CDSF conditions followed by standard conditions were transplanted. The rectangle indicates the area shown in the inset. Bar, 1cm. The inset shows an enlarged image of a tiny mass of the mESCs depicted by dashed lines with a scale bar of 0.1 cm. See supporting information Fig. S2 for detail. (TIF) [file pone.0021355.s001.tif]

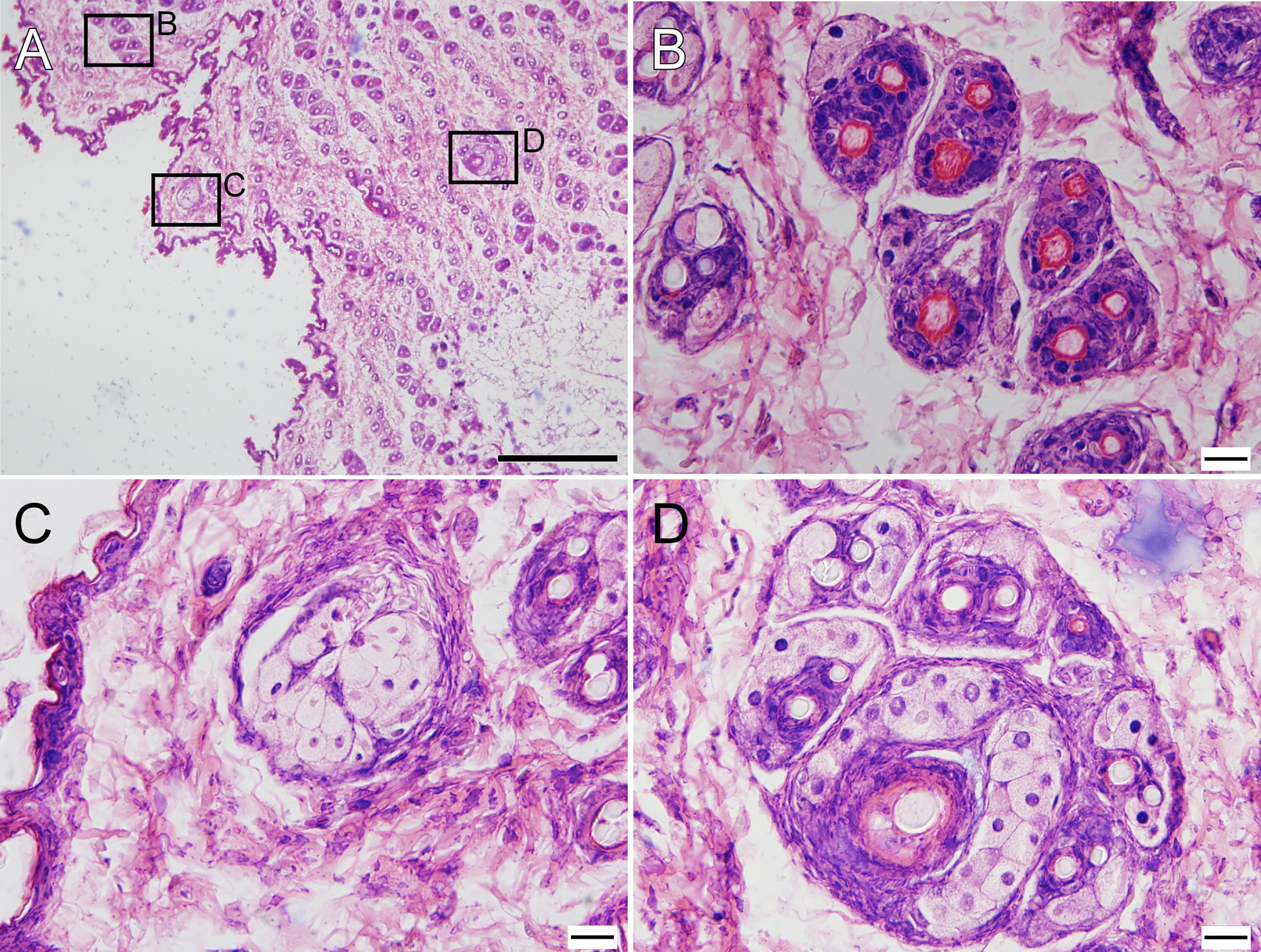

Supplement: Figure S2 — Mouse embryonic stem cells exhibited initial differentiation as early as one week after transplantation when cultured under CDSF-Standard conditions. Mouse ESCs were cultured in CDSF for three passages followed by transfer to standard conditions for two passages prior to transplantation. (A): An epithelialized cellular mass has aggregates formed among the collagen fibers (stained pale pink) used to transplant the mESCs. Rectangles indicate the areas shown in B, C and D. Bar, 500 µm. (B-D): Two types of cells are prominent, one of which is reminiscent of keratin pearls (B and D), and the other that resembles cartilage (C and D). Bars, 20 µm. (TIF) [file pone.0021355.s002.tif]

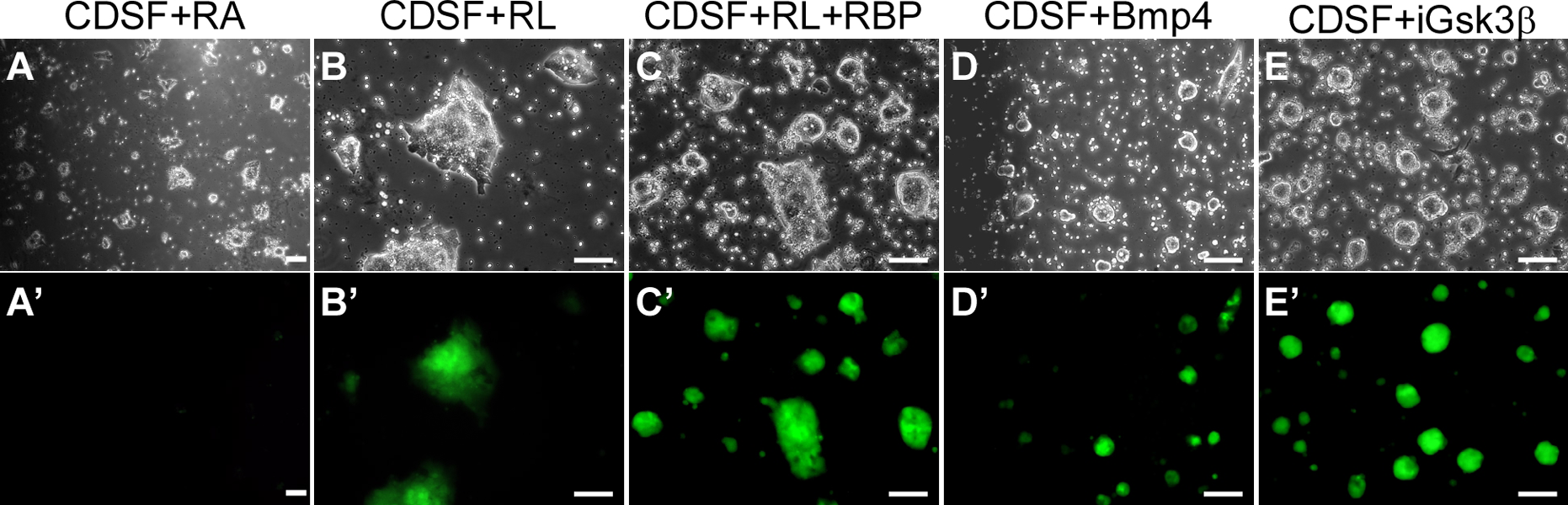

Supplement: Figure S3 — Identification of factors that support the tumor-like growth of mouse embryonic stem cells maintained under CDSF conditions. (A-E): Phase contrast (top) and fluorescence (bottom) images of mESCs under CDSF conditions supplemented with each factor indicated above are shown. Bars, 20 µm. CDSF+RA, CDSF with retinoic acid; CDSF+RL, CDSF with retinol; CDSF+RL+RBP, CDSF with retinol and retinol binding protein; CDSF+Bmp4, CDSF with Bmp4; CDSF+iGsk3β, CDSF with the Gsk3β inhibitor. (TIF) [file pone.0021355.s003.tif]

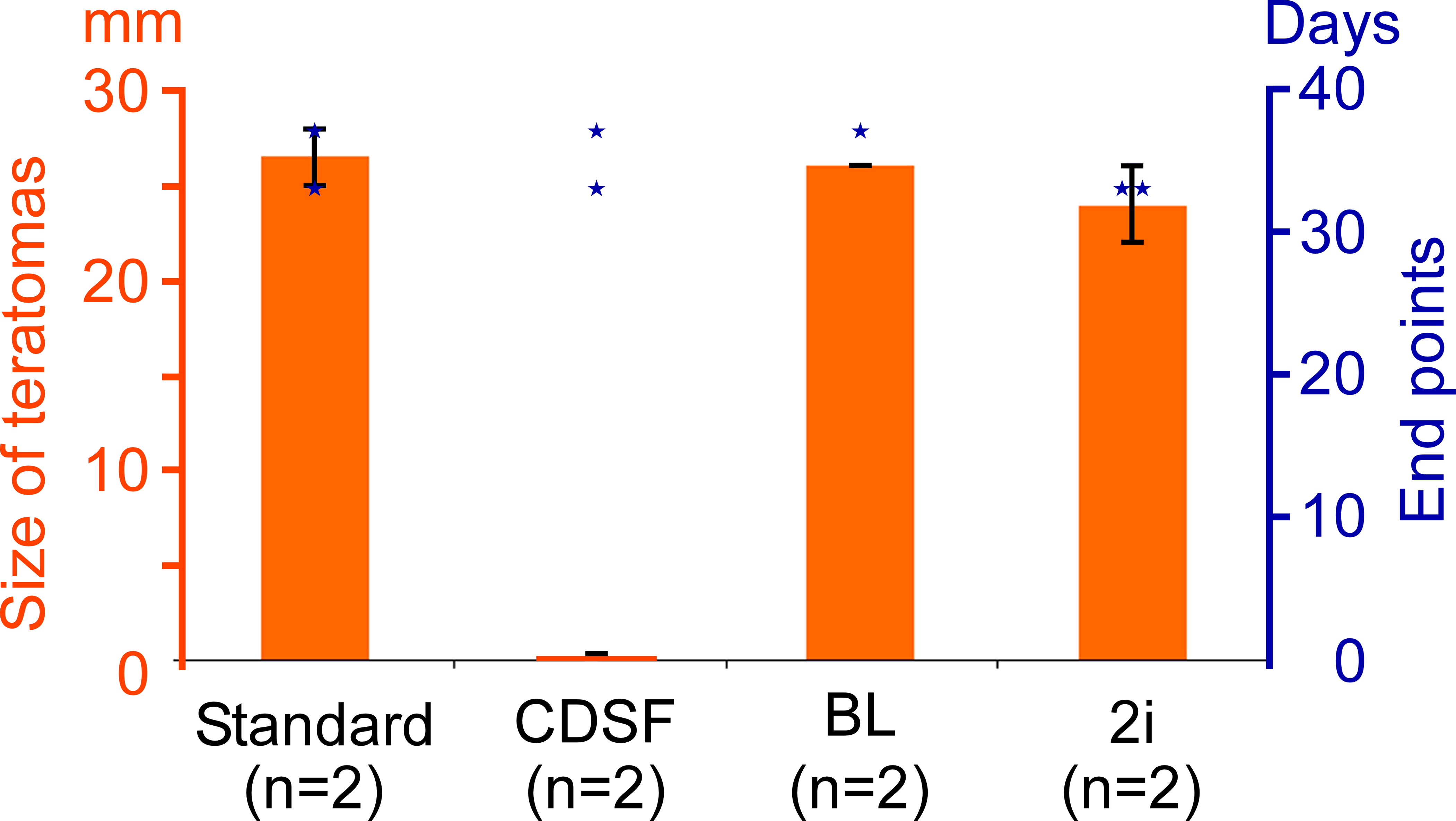

Supplement: Figure S4 — The incidence of teratoma formation was compared among different CDSF conditions. The sizes of the teratomas formed (orange bar, left axis) and the number of days required for the experimental NOD-SCID mice to reach their end points (blue stars, right axis) were compared among mESCs cultured under the conditions indicated. Parentheses indicate the number of biological replicates (i.e., mESCs prepared at different passages) per culture condition. Standard errors of the means are indicated by bars. Two transplantations for ESF7 showed no sign of teratoma formation when paired with the standard conditions. Standard, Standard conditions; CDSF, CDSF conditions; BL, other established CDSF conditions supplemented with N2, B27, Bmp4 and LIF; 2i, other established CDSF conditions supplemented with N2, B27, and pharmacological inhibitors of Erk and Gsk3β. (TIF) [file pone.0021355.s004.tif]

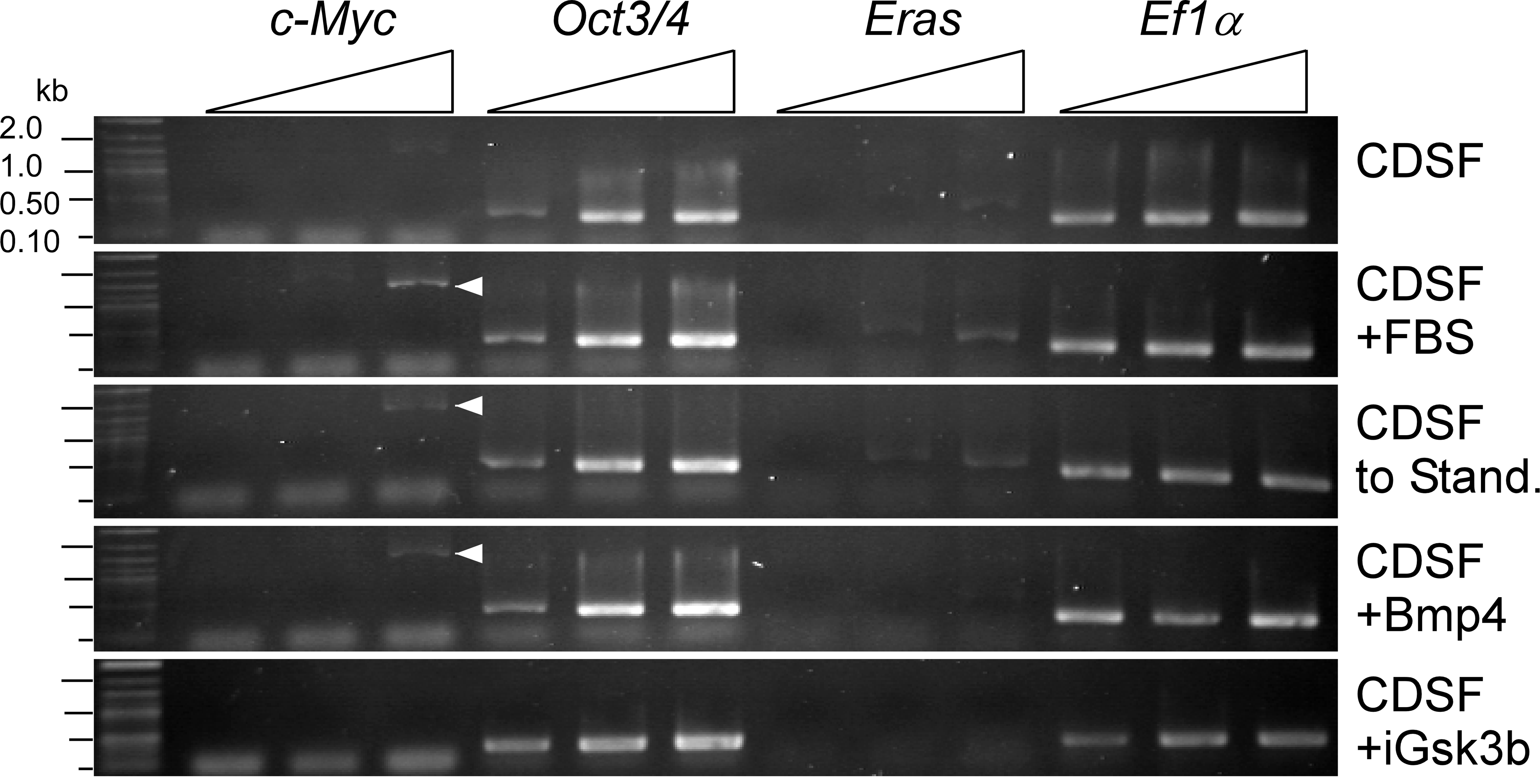

Supplement: Figure S5 — Expression levels of markers were compared among different culture conditions. Abundance of each transcript indicated above was examined in mESCs cultured under each condition on the right by 19, 21 and 23 cycles of PCR (indicated by triangles). Ef1α was used as a reference. White arrowheads indicate the PCR product of c-Myc. (TIF) [file pone.0021355.s005.tif]
