# Supplementary material for: Short-Term Serum-Free Culture Reveals that Inhibition of Gsk3β Induces the Tumor-Like Growth of Mouse Embryonic Stem Cells
Source: PLoS One. 2011 Jun 23;6(6):e21355. doi: 10.1371/journal.pone.0021355 (PMC3121758; doi:10.1371/journal.pone.0021355)
Supplement: Table S1 — Effects of serum on the tumorigenicity of mouse embryonic stem cells. (DOCX) [file pone.0021355.s006.docx]

**Table S1. Effects of serum on the tumorigenicity of mouse embryonic stem cells.**

| **Conditions** | **# of Biological replicates** | **Teratomas formed** |  |
| --- | --- | --- | --- |
| **Series I** |  |  |  |
| CDSF | 7 | 0 | *p*=0.0001554^a^ |
| With FBS | 8 | 8 |  |
| CDSF+FBS | 4 | 4 |  |
| CDSF to Stand. | 4 | 4 |  |
| **Series II** |  |  |  |
| With FBS (Standard) | 2 | 2 |  |
| CDSF (ESF7) | 2 | 0 |  |
| CDSF (N2B27-BL) | 2 | 1 |  |
| CDSF (N2B27-2i) | 2 | 2 |  |

^a^: Compared with conditions “with FBS”.
